# Impacto do Alto Risco Cardiovascular na Mortalidade Hospitalar em Pacientes Internados em Terapia Intensiva por COVID-19

**DOI:** 10.36660/abc.20210349

**Published:** 2022-05-04

**Authors:** Bruno Ferraz de Oliveira Gomes, João Luiz Fernandes Petriz, Iliana Regina Ribeiro Menezes, Anny de Sousa Azevedo, Thiago Moreira Bastos da Silva, Valdilene Lima Silva, Leticia de Sousa Peres, David Fernandes Pedro Pereira, Giovanni Possamai Dutra, Suzanna Andressa Morais de Paula, Bárbara Ferreira da Silva Mendes, Plinio Resende do Carmo, Basilio de Bragança Pereira, Gláucia Maria Moraes de Oliveira

**Affiliations:** 1 Barra D’Or Hospital Rio de Janeiro RJ Brasil Barra D’Or Hospital, Rio de Janeiro, RJ – Brasil; 2 Universidade Federal do Rio de Janeiro Rio de Janeiro RJ Brasil Universidade Federal do Rio de Janeiro, Rio de Janeiro, RJ – Brasil; 3 Universidade Federal do Rio de Janeiro Instituto do Coração Edson Saad Rio de Janeiro RJ Brasil Universidade Federal do Rio de Janeiro – ICES Instituto do Coração Edson Saad, Rio de Janeiro, RJ – Brasil

**Keywords:** Doenças Cardiovasculares/complicações, COVID-19, Terapia Intensiva, Troponina Ultrassensível, Lesão miocárdica, Risco Cardiovascular, Pacientes Internados

## Abstract

**Fundamento:**

Alguns estudos demonstraram uma maior prevalência de óbitos em portadores de fatores de risco cardiovascular (FRC) durante internação por COVID-19.

**Objetivos:**

Avaliar o impacto do alto risco cardiovascular em pacientes internados em terapia intensiva por COVID-19

**Métodos:**

Estudo retrospectivo com pacientes admitidos em terapia intensiva, com diagnóstico confirmado de COVID-19 por RT-PCR e com pelo menos uma dosagem de troponina durante a internação. Os critérios para definição de paciente de alto risco cardiovascular (ARC) foram: histórico de doença cardiovascular estabelecida (infarto, AVC ou doença arterial periférica), diabetes, doença renal crônica com clearance < 60ml/min ou presença de 3 FRC (hipertensão, tabagismo, dislipidemia ou idade > 65 anos). O desfecho primário deste estudo é mortalidade hospitalar por todas as causas. P<0,05 foi considerado significativo.

**Resultados:**

Foram incluídos 236 pacientes, média de idade= 61,14±16,2 anos, com 63,1% homens, 55,5% hipertensos e 33,1% diabéticos. Um total de 47,4% dos pacientes apresentavam ARC. Observou-se um aumento significativo da mortalidade conforme aumento do número de fatores de risco (0 FRC: 5,9%; 1 FRC: 17,5%; 2 FRC: 32,2% e ≥3 FRC: 41,2%; p=0,001). Na regressão logística ajustada para gravidade (escore SAPS3), o grupo de alto risco cardiovascular e troponina elevada apresentou maior ocorrência de mortalidade hospitalar (OR 40,38; IC95% 11,78-138,39). Pacientes sem alto risco cardiovascular, mas com troponina elevada, também exibiram associação significativa com o desfecho primário (OR 16,7; IC95% 4,45-62,74).

**Conclusão:**

Em pacientes internados em terapia intensiva por COVID-19, a presença de alto risco cardiovascular afeta a mortalidade hospitalar somente em pacientes que apresentaram elevação de troponina.

## Introdução

Desde dezembro de 2019, observamos um crescimento expressivo do número de casos da doença causada pelo novo coronavírus (COVID-19) que motivou a declaração de pandemia em março de 2020. Até o presente momento, mais de 100 milhões de pessoas já foram infectadas, provocando mais de 2 milhões de mortes em todo o mundo.^1^

Estudos iniciais que avaliaram pacientes internados por COVID-19 identificaram maior vulnerabilidade dos pacientes portadores de fatores de risco cardiovascular.^2 , 3^ Nessa população, a elevação de troponina, que se mostrou marcador independente de morte, é mais prevalente.^4^ Em pacientes admitidos em unidade de terapia intensiva, essa mortalidade é ainda maior.^5^

Por outro lado, a maioria dos estudos publicados sobre essa temática foram realizados em países desenvolvidos, onde encontramos uma maior prevalência desses fatores de risco.^6^ Dessa forma, dados sobre o desfecho desses pacientes em países em desenvolvimento são necessários.

O objetivo deste estudo é avaliar a mortalidade hospitalar em pacientes internados em terapia intensiva por COVID-19 conforme o risco cardiovascular.

## Métodos

### População de estudo

Estudo retrospectivo em que foram incluídos pacientes admitidos em unidade de terapia intensiva de hospital terciário, com quadro clínico compatível de COVID-19 e confirmação sorológica por meio de RT-PCR, e com pelo menos uma dosagem de troponina durante a internação (amostra de conveniência). O período de estudo foi de março/2020 a maio/2020. Foram excluídos pacientes portadores de demência, doenças avançadas/terminais, pacientes em tratamento paliativo e com permanência hospitalar inferior a 2 dias.

Os dados foram obtidos por meio de consulta a prontuário eletrônico, em que todas as evoluções foram checadas em busca das informações pertinentes. Os dados coletados foram: idade, gênero, troponina de admissão e pico, d-dímero de admissão e pico, obesidade (IMC ≥ 30kg/m^2^ ), insuficiência cardíaca prévia (relato de quadro clínico compatível, ecocardiograma com fração de ejeção de reduzida, ou uso de medicamentos para tratamento de insuficiência cardíaca), insuficiência renal (clearance de creatinina < 60ml/min), infarto agudo do miocárdio (IAM) prévio, acidente vascular encefálico (AVC) prévio, doença arterial periférica, tabagismo e dislipidemia.

Os critérios para definição de paciente de alto risco cardiovascular (ARC) foram: história de doença cardiovascular estabelecida (infarto, AVC ou doença arterial periférica), diabetes, doença renal crônica com clearance < 60ml/min ou presença de 3 fatores de risco (hipertensão, tabagismo, dislipidemia ou idade > 65 anos).

O kit de troponina utilizado é comercializado pela VITROS® Ortho Clinical Diagnostics, com ponto de corte de 9ng/L (percentil 99). Acima desse valor, considera-se que troponina está elevada.

O desfecho primário deste estudo é mortalidade hospitalar por todas as causas enquanto o desfecho secundário deste estudo é composto por morte hospitalar, ocorrência de injúria miocárdica e necessidade de ventilação mecânica.

### Análise estatística

As variáveis contínuas foram apresentadas por média e desvio-padrão (quando houve distribuição normal) ou mediana e intervalo interquartil (quando não houve distribuição normal). O teste de normalidade utilizado foi o Kolmogorov-Smirnov. As variáveis categóricas foram expressas em percentual. As variáveis clínicas e laboratoriais foram comparadas conforme desfecho primário e secundário em análise univariada por meio do teste de qui-quadrado (variáveis categóricas) e teste t de Student não-pareado ou teste não paramétrico Mann-Whitney (variáveis contínuas). Os desfechos também foram avaliados conforme o número de fatores de risco cardiovascular e em 4 subgrupos: (ARC com troponina elevada, ARC com troponina normal, não-ARC com troponina elevada e não-ARC com troponina normal). Esses subgrupos também foram avaliados por regressão logística binomial ajustada para gravidade (escore SAPS3) para o desfecho primário. Por fim, todas variáveis estudadas foram incluídas na árvore de classificação,^7^ um método de aprendizado de máquina, visando identificar variáveis preditivas do desfecho primário. P<0,05 foi considerado significativo. Para análise estatística, foi utilizado o programa SPSS versão 26.

### Aspectos éticos

Este estudo foi aprovado pelo comitê de ética do Instituto D’Or de Ensino e Pesquisa e está registrado na plataforma Brasil sob o número 33206620.0.0000.5249. Por ser um estudo retrospectivo, o termo de consentimento informado foi dispensado pelo comitê de ética.

## Resultados

**Figura 1:**
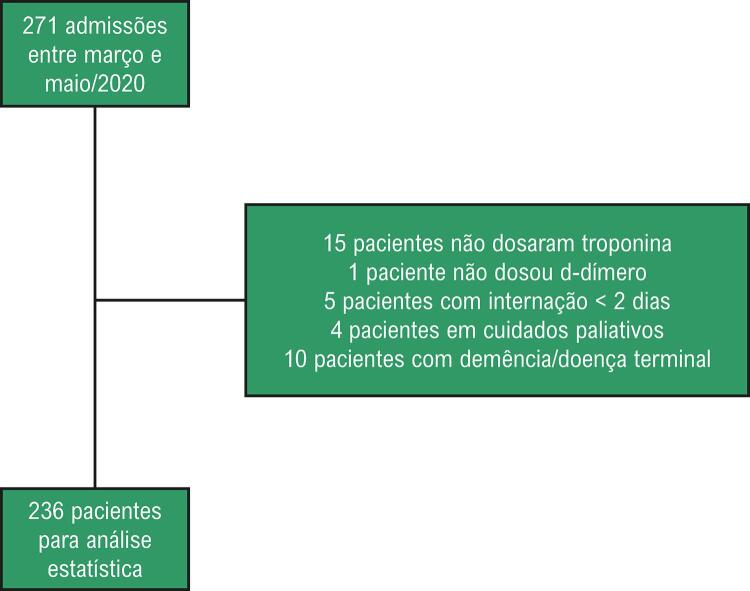
– Fluxo de inclusão de pacientes no estudo.

O fluxo de inclusão dos pacientes no estudo está exposto na figura 1 . Após avaliação de 271 admissões, foram incluídos 236 pacientes para análise.

As características dessa população estão expostas na tabela 1 .

**Tabela 1:** – Características da amostra estudada

Características	N=236
Idade (anos) - média ± DP	61,14 ± 16,2
Idade ≥ 65 anos (%)	45,3
Sexo masculino (%)	63,1
Obesidade (%)	20,3
IC prévia (%)	4,2
DRC (%)	5,1
HAS (%)	55,5
Diabetes (%)	33,1
IAM prévio (%)	5,9
Doença arterial periférica (%)	8,9
Fibrilação atrial (%)	3,0
AVE prévio (%)	3,4
Tabagismo (%)	4,7
Dislipidemia (%)	13,6
Ventilação mecânica (%)	30,4
Uso de vasopressores (%)	25,0
Terapia de substituição renal (%)	10,7
Elevação de troponina (%)	29,7
SAPS3 – mediana (IIQ)	42,0 (34,5 – 50,0)
Tempo de internação (dias) – mediana (IIQ)	7 (4 - 14)
Desfecho primário (%)	24,2
Desfecho secundário (%)	38,6

*DP: desvio-padrão; IC: insuficiência cardíaca; DRC: doença renal crônica; HAS: hipertensão arterial sistêmica; IAM: infarto agudo do miocárdio; AVE: acidente vascular encefálico; IIQ: intervalo interquartil.*

**Figura 2:**
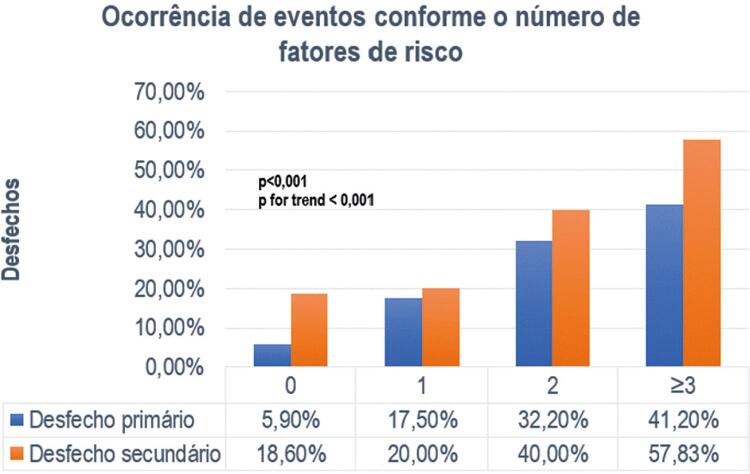
– Evolução do desfecho primário e secundário conforme o número de fatores de risco, realizada pelo teste qui-quadrado.

Foi observada alta prevalência de hipertensão arterial (55,5%) e diabetes (33,1%). Os demais fatores de risco foram menos prevalentes. Na análise da mortalidade conforme o número de fatores de risco, encontramos maior ocorrência do desfecho primário e secundário em pacientes com mais fatores de risco cardiovascular ( figura 2 ).

Na tabela 2 , está exposta a análise univariada das variáveis clínicas e fatores de risco conforme ocorrência de desfecho primário.

**Tabela 2:** – Análise univariada das características conforme desfecho primário

Variáveis	Desfecho primário		p

	Sim (n=57)	Não (n=179)	
Idade (anos) - média ± DP	71,3±13,5	59,2±15,9	<0,001
Idade ≥ 65 anos (%)	75,4	35,8	<0,001
Obesidade (%)	28,1	17,9	0,072
IC prévia (%)	10,5	2,2	0,015
DRC (%)	12,3	2,8	0,010
HAS (%)	68,4	51,4	0,017
Diabetes (%)	49,1	27,9	0,003
IAM prévio (%)	7,0	5,6	0,450
Doença arterial periférica (%)	10,5	8,4	0,396
Fibrilação atrial (%)	8,8	1,1	0,010
AVE prévio (%)	8,8	1,7	0,022
Tabagismo (%)	7,0	3,9	0,260
Dislipidemia (%)	15,8	12,8	0,357
Ventilação Mecânica (%)	87,0	12,4	<0,001
Uso de vasopressores (%)	70,4	10,6	<0,001
TRS (%)	35,2	2,9	<0,001
Elevação de troponina (%)	80,7	13,4	<0,001
Pico de troponina (ng/L) - mediana (IIQ)	58 (13-276)	7 (4 – 10)	<0,001
Pico de D-dímero (ng/mL) - mediana (IIQ)	7.857 (4.124-24.121)	1.327 (754-3.087)	<0,001
SAPS3 - mediana (IIQ)	52 (44 - 61)	39 (34 - 46)	<0,001
Risco Cardiovascular alto (%)	70,2	40,2	<0,001

*Comparação das características clínicas e laboratoriais conforme o desfecho primário (morte hospitalar). DP: desvio-padrão; IC: insuficiência cardíaca; DRC: doença renal crônica; HAS: hipertensão arterial sistêmica; IAM: infarto agudo do miocárdio; AVE: acidente vascular encefálico; TRS: terapia de substituição renal; IIQ: intervalo interquartil.*

Na análise univariada, diversas características clínicas estiveram associadas significativamente à maior prevalência do desfecho primário. A tabela 3 expõe a análise univariada relacionada ao desfecho secundário.

**Tabela 3:** – Análise univariada das características conforme desfecho secundário

Variáveis	Desfecho secundário		p

	Sim (n=86)	Não (n=138)	
Idade (anos) - média ± DP	69,0±15,5	57,8±15,1	<0,001
Idade ≥ 65 anos (%)	67,0	31,7	<0,001
Obesidade (%)	23,1	18,6	0,253
IC prévia (%)	7,7	2,1	0,041
DRC (%)	9,9	2,1	0,010
HAS (%)	69,2	46,9	0,001
Diabetes (%)	40,7	28,3	0,034
IAM prévio (%)	8,8	4,1	0,118
Doença arterial periférica (%)	12,1	6,9	0,130
Fibrilação atrial (%)	5,5	1,4	0,080
AVE prévio (%)	6,6	1,4	0,039
Tabagismo (%)	5,5	4,1	0,427
Dislipidemia (%)	14,3	13,1	0,471
Uso de vasopressores (%)	62,8	1,4	<0,001
TRS (%)	27,9	0,0	<0,001
Pico de D-dímero (ng/mL) - mediana (IIQ)	6.118 (3.365-18.433)	1.030 (613-1.880)	<0,001
SAPS3 - mediana (IIQ)	37 (29-43)	50 (43-60)	<0,001
Risco cardiovascular alto (%)	62,6	37,9	<0,001

*Comparação das características clínicas e laboratoriais conforme o desfecho secundário (composto por morte hospitalar, injúria miocárdica e necessidade de ventilação mecânica). DP: desvio-padrão; IC: insuficiência cardíaca; DRC: doença renal crônica; HAS: hipertensão arterial sistêmica; IAM: infarto agudo do miocárdio; AVE: acidente vascular encefálico; TRS: terapia de substituição renal; IIQ: intervalo interquartil.*

**Figura 3:**
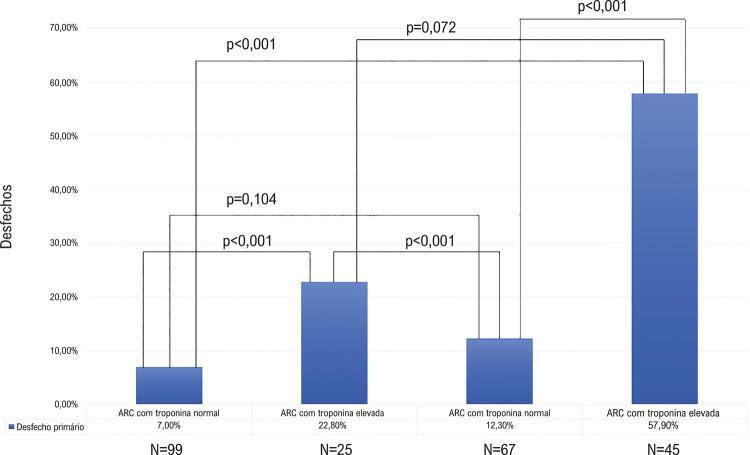
– Ocorrência do desfecho primário nos subgrupos determinados conforme risco cardiovascular e elevação de troponina. Comparação entre grupos realizada com o teste de qui-quadrado. ARC: alto risco cardiovascular.

Semelhantemente ao desfecho primário, diversas características exibiram associação com o desfecho secundário. Na análise conforme grupo de risco (ARC com troponina elevada, ARC com troponina normal, não-ARC com troponina elevada e não-ARC com troponina normal), observamos que o grupo de ARC com troponina positiva apresentou maior mortalidade (57,9%), significativamente superior aos grupos com troponina normal, mas sem diferença estatística em relação ao grupo não-ARC com troponina positiva ( Figura 3 ).

Na regressão logística ajustada para gravidade (escore SAPS3), o grupo de pacientes com ARC e troponina positiva apresentou o maior risco de mortalidade, seguido do grupo não-ARC com troponina positiva ( Tabela 4 )

**Tabela 4:** – Regressão logística binomial para o desfecho primário.

Variável	OR	IC 95%	p
não-ARC com troponina normal	Referência		
não-ARC com troponina elevada	16,70	4,45-62,74	<0,001
ARC com troponina normal	2,06	0,56-7,56	0,2745
ARC com troponina elevada	40,38	11,78-138,39	<0,001
SAPS3	1,05	1,02-1,09	0,0023

*ARC: alto risco cardiovascular.*

**Figura 4:**
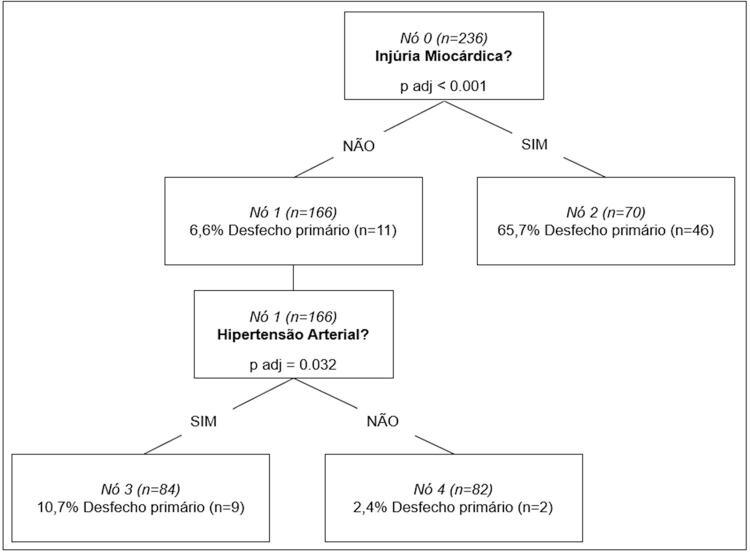
– Árvore de Classificação para o desfecho primário.

Na árvore de classificação, para o desfecho primário, encontramos a troponina positiva como primeira característica classificadora, seguida de hipertensão arterial. Esse modelo de classificação apresentou acurácia de 85,2% ( Figura 4 ).

## Discussão

Este estudo avaliou o impacto do alto risco cardiovascular em pacientes internados em terapia intensiva por COVID-19. Essa abordagem permitiu a inclusão de pacientes com perfil de gravidade mais elevado e maior prevalência de fatores de risco cardiovasculares. Nessa população, mais da metade dos pacientes eram portadores de hipertensão arterial, e um terço, de diabetes. Ressalta-se também a elevada proporção de pacientes com idade superior a 65 anos (45,3%). O principal achado deste estudo foi a observação que pacientes de alto risco cardiovascular apresentaram mortalidade significativamente maior somente quando associado à troponina elevada.

A maioria dos estudos publicados avaliaram somente o impacto dos fatores de risco cardiovasculares na mortalidade por COVID-19, gerando resultados conflitantes. Di Castelnuovo et al.,^8^ estudaram quase 4000 pacientes em um estudo multicêntrico italiano, utilizando técnicas de análise estatística baseadas em aprendizado de máquina. Esse estudo incluiu pacientes mais idosos (54,8% acima de 65 anos), porém com prevalência similar de hipertensão arterial (48,8%) e menor prevalência de diabetes (19%). Os principais preditores de morte hospitalar foram disfunção renal, níveis elevados de PCR e idade avançada. Não foi encontrada associação com obesidade, tabagismo, doença cardiovascular e comorbidades relacionadas.

Collard et al.,^9^ analisaram dados de oito hospitais participantes da coorte CovidPredict na Alemanha.^9^ Para análise dos fatores de risco cardiovasculares, eles avaliaram o uso de anti-hipertensivos, antidiabéticos e hipolipemiantes. O estudo incluiu 1604 pacientes com idade média de 66 anos, sendo 46% hipertensos e 25,7% diabéticos. Foi observado que pacientes com mais de um fator de risco cardiovascular apresentaram mortalidade em 3 semanas 52% superior, independentemente de gênero e idade. Além disso, o uso de dois ou mais anti-hipertensivos, ou antidiabéticos, ou um hipolipemiante esteve associado ao pior prognóstico em pacientes com COVID-19. Nosso estudo encontrou resultado semelhante, demonstrando aumento progressivo na mortalidade hospitalar conforme o aumento do número de fatores de risco.

Silverio et al.,^10^ realizaram uma meta-análise que incluiu 18300 pacientes.^10^ Na análise univariada, foi observada associação de morte hospitalar com idade, diabetes e hipertensão. No entanto, na regressão multivariada, apenas diabetes e idade mais avançada foram associadas a morte hospitalar.

Apenas um trabalho utilizou estratégia de análise de dados similar ao presente estudo. Guo et al.,^11^ analisaram 187 pacientes em Wuhan (origem da pandemia), como objetivo de avaliar a associação de doença cardiovascular subjacente e injúria miocárdica com desfechos fatais em pacientes com COVID-19.^11^ Essa população era mais jovem (idade média=58,5 anos), com menor prevalência de hipertensão (32,6%) e diabetes (15,0%). Na análise estatística desse artigo, não foram avaliados os dados relacionados ao risco cardiovascular, mas quanto à doença cardiovascular estabelecida, definida pela presença de hipertensão, doença arterial coronariana e cardiomiopatia. Os pacientes com doença cardiovascular estabelecida e troponina elevada apresentaram mortalidade de 69,44%, enquanto os pacientes sem doença cardiovascular, mas com troponina elevada apresentaram mortalidade de 37,5%. Esses resultados são semelhantes aos descritos por este artigo, apesar de terem sido utilizados um critério de classificação e técnicas estatísticas diferentes.

Dessa forma, observamos que nenhum estudo publicado até o momento teve como objetivo o estudo do paciente caracterizado como alto risco cardiovascular, que representou 47,4% desta amostra. A troponina, marcador de injúria miocárdica, demonstrou sua importância prognóstica em estudos prévios,^4 , 11^ e neste estudo, na árvore de classificação, foi o primeiro marcador prognóstico de mortalidade hospitalar. No subgrupo de pacientes que não apresentou injúria miocárdica, a presença de hipertensão arterial foi a comorbidade significativamente associada à morte hospitalar.

Na regressão logística, foi utilizado o escore SAPS3, um escore de gravidade em terapia intensiva realizado à admissão,^12^ para ajustar potenciais confundidores na análise dos subgrupos conforme risco cardiovascular e elevação de troponina. Após ajuste, observou-se que pacientes com alto risco cardiovascular e troponina elevada apresentaram risco 40 vezes superior de morte hospitalar em relação ao paciente sem alto risco e troponina normal, independentemente da gravidade apresentada à admissão. Em pacientes com troponina elevada, porém sem alto risco cardiovascular, foi observado elevado risco de mortalidade (OR 16,70; IC95% 4,45-62,74), porém com menor magnitude em relação aos pacientes de alto risco cardiovascular e injúria miocárdica. Em contrapartida, nos pacientes sem injúria miocárdica, o alto risco cardiovascular não implicou significativamente em morte hospitalar.

Este estudo apresenta algumas limitações que são inerentes a um estudo retrospectivo. Todos os dados foram avaliados pela verificação de prontuário eletrônico, não sendo possível a confirmação de dados, ou perguntas adicionais ao paciente ou familiares. Além disso, nem todos os pacientes realizaram ecocardiograma ou dosagem de BNP, informações importantes em um estudo que avalia impacto cardiovascular da COVID-19. Ademais, o número reduzido de pacientes no estudo limita a análise estatística e as conclusões a partir desses resultados.

Apesar das limitações, este é o primeiro estudo que analisa especificamente a população de alto risco cardiovascular em pacientes admitidos em unidade de terapia intensiva por COVID-19.

## Conclusões

Em pacientes internados em terapia intensiva por COVID19, a presença de alto risco cardiovascular tem impacto na mortalidade hospitalar somente em pacientes que apresentaram elevação de troponina.
