# Supplementary material for: Impacto do Alto Risco Cardiovascular na Mortalidade Hospitalar em Pacientes Internados em Terapia Intensiva por COVID-19
Source: Arq Bras Cardiol. 2022 May 4;118(5):927–34. [Article in Portuguese] doi: 10.36660/abc.20210349 (PMC9368869; doi:10.36660/abc.20210349)
Supplement: Translation

# Translation: Impact of High Cardiovascular Risk on Hospital Mortality in Intensive Care Patients Hospitalized for COVID-19

**Authors:** Bruno Ferraz de Oliveira Gomes, João Luiz Fernandes Petriz, Iliana Regina Ribeiro Menezes, Anny de Sousa Azevedo, Thiago Moreira Bastos da Silva, Valdilene Lima Silva, Leticia de Sousa Peres, David Fernandes Pedro Pereira, Giovanni Possamai Dutra, Suzanna Andressa Morais de Paula, Bárbara Ferreira da Silva Mendes, Plinio Resende do Carmo, Basilio de Bragança Pereira, Gláucia Maria Moraes de Oliveira

**Affiliations:** 1 Brasil Barra D’Or Hospital, Rio de Janeiro, RJ – Brasil; 2 Brasil Universidade Federal do Rio de Janeiro, Rio de Janeiro, RJ – Brasil; 3 Brasil Universidade Federal do Rio de Janeiro – ICES Instituto do Coração Edson Saad, Rio de Janeiro, RJ – Brasil

**Keywords:** Cardiovascular Diseases/complications, COVID-19, Intensive Care, Ultrasensitivity Troponin, Myocardial Injury, Cardiovascular Risk, Inpatients

## Abstract

**Background:**

Some studies have shown a higher prevalence of deaths in patients with cardiovascular risk factors (CRF) during hospitalization for COVID-19.

**Objectives:**

To assess the impact of high cardiovascular risk in patients hospitalized in intensive care for COVID-19

**Methods:**

Retrospective study with patients admitted to an intensive care unit, with a diagnosis of COVID-19 confirmed by RT-PCR, and with at least one troponin measurement during hospitalization. The criteria for defining high cardiovascular risk (HCR) patients were: history of established cardiovascular disease (myocardial infarction, stroke, or peripheral arterial disease), diabetes, chronic kidney disease with clearance < 60ml/min, or presence of 3 CRFs (hypertension, smoking, dyslipidemia, or age > 65 years). The primary outcome of this study is all-cause in-hospital mortality. P<0.05 was considered significant.

**Results:**

This study included 236 patients, mean age = 61.14±16.2 years, with 63.1% men, 55.5% hypertensive, and 33.1% diabetic; 47.4% of the patients also presented HCR. A significant increase in mortality was observed as the number of risk factors increased (0 FRC: 5.9%; 1 FRC: 17.5%; 2 FRC: 32.2% and ≥3 FRC: 41.2%; p=0.001). In the logistic regression adjusted for severity (SAPS3 score), the HCR and myocardial injury group had a higher occurrence of in-hospital mortality (OR 40.38; 95% CI 11.78-138.39). Patients without HCR but with myocardial injury also exhibited a significant association with the primary outcome (OR 16.7; 95% CI 4.45-62.74).

**Conclusion:**

In patients hospitalized in intensive care for COVID-19, HCR impacts in-hospital mortality only in patients with myocardial injury.

## Introduction

Since December 2019, we have observed a significant rise in the number of cases of disease caused by the new coronavirus (COVID-19), which led to the declaration of a pandemic in March 2020. To date, more than 100 million people have been infected, causing more than 2 million deaths worldwide.^1^

Initial studies that evaluated patients hospitalized for COVID-19 identified greater vulnerability of patients with cardiovascular risk factors.^2 , 3^ In this population, myocardial injury documented by troponin increase, which proved to be an independent marker of death, is more prevalent.^4^ In patients admitted to the intensive care unit, this mortality is even higher.^5^

By contrast, most studies published on this topic were carried out in developed countries, where we found a higher prevalence of these risk factors.^6^ Therefore, data on the outcome of these patients in developing countries are needed.

The aim of this study is to assess hospital mortality in intensive care patients hospitalized for COVID-19 according to cardiovascular risk.

## Methods

### Study population

This work was a retrospective study including patients admitted to the intensive care unit of a tertiary hospital for COVID-19, with a serological confirmation by means of RT-PCR and with at least one ultrasensitive troponin measurement during hospitalization (convenience sample). The study period was from March/2020 to May/2020. Patients with dementia, advanced/terminal illnesses, patients undergoing palliative care, and those with a hospital stay of fewer than two days were excluded.

Data were obtained by consulting hospital electronic medical records. The data collected were: age, gender, admission and peak of ultrasensitive troponin, admission and peak d-dimer, obesity (BMI ≥ 30kg/m^2^ ), previous heart failure (report of previous signs and symptoms compatible with heart failure or echocardiogram with reduced ejection fraction or use of medications to treat heart failure), renal failure (creatinine clearance < 60ml/min), previous acute myocardial infarction (AMI), previous stroke, peripheral arterial disease, smoking, and dyslipidemia.

The criteria for defining high cardiovascular risk (HRC) patients were: history of established cardiovascular disease (infarction, stroke, or peripheral arterial disease), diabetes, chronic kidney disease with clearance < 60ml/min, or presence of three risk factors (hypertension, smoking, dyslipidemia, or age > 65 years).

The ultrasensitive troponin kit used in the study was provided by VITROS® Ortho Clinical Diagnostics, with a cutoff point of 9ng/L (99th percentile). Above this value, we considered myocardial injury.

The primary outcome of this study is all-cause in-hospital mortality, while the secondary outcome is composite of in-hospital mortality, myocardial injury, and need for mechanical ventilation support.

### Statistical analysis

Continuous variables were presented as mean and standard deviation (when there is a normal distribution) or median and interquartile range (not normally distributed). The normality test used was the Kolmogorov-Smirnov. Categorical variables were displayed as a percentage. Clinical and laboratory variables were compared according to primary and secondary outcomes in univariate analysis using the chi-square test (categorical variables) and unpaired Student’s t-test or non-parametric Mann-Whitney test (continuous variables). Outcomes were also assessed according to the number of cardiovascular risk factors and were divided into four subgroups: (HCR with myocardial injury, HCR without myocardial injury, non-HCR with myocardial injury, and non-HCR without myocardial injury). These subgroups were also evaluated using binomial logistic regression adjusted by severity (using SAPS3 score) for the primary outcome. Finally, all variables studied were included in the classification tree,^7^ a machine learning method, aiming to identify predictive variables of the primary outcome. P<0.05 was considered significant. For statistical analysis, the SPSS, version 26, was used.

### Ethical aspects

This study was approved by the ethics committee of Instituto D’Or de Ensino e Pesquisa and is registered on the Brasil platform under number 33206620.00.0000.5249. As it is a retrospective study, the informed consent form was waived by the ethics committee.

## Results

The flow chart of the inclusion of patients in the study is shown in Figure 1 . After evaluating 271 admissions, 236 patients were included for analysis.

**Figure 1:** – Flow chart of inclusion of patients in the study.

The characteristics of this population are shown in Table 1 .

**Table 1:** – General characteristics of the studied sample

Variables	N=236
Age (years) - mean ± SD	61.14 ± 16.2
Age ≥ 65 years (%)	45.3
Male sex (%)	63.1
Obesity (%)	20.3
Previous HF (%)	4.2
CKD (%)	5.1
SAH (%)	55.5
Diabetes (%)	33.1
Previous AMI (%)	5.9
PAD (%)	8.9
AF (%)	3.0
Previous stroke (%)	3.4
Tobacco use (%)	4.7
Dyslipidemia (%)	13.6
Mechanical ventilation (%)	30.4
Vasopressor use (%)	25.0
Renal replacement therapy (%)	10.7
Myocardial injury (%)	29.7
SAPS3 – median (IQR)	42.0 (34.5 – 50.0)
Hospitalization duration (days) – median (IQR)	7 (4 - 14)
Primary outcome (%)	24.2
Secondary outcome (%)	38.6

*SD: standard deviation; HF: heart failure; CKD: chronic kidney disease; SAH: systemic arterial hypertension; AMI: acute myocardial infarction; PAD: Peripheral Artery Disease; AF: atrial fibrillation; IQR: interquartile range.*

There was a high prevalence of arterial hypertension (55.5%) and diabetes (33.1%). The other risk factors were less prevalent. In the analysis of mortality according to the number of risk factors, we found a higher occurrence of the primary and secondary outcomes in patients with more cardiovascular risk factors ( Figure 2 ).

**Figure 2:** – Evolution of the primary and secondary outcome according to the number of risk factors, performed using the chi-square test.

Table 2 shows the univariate analysis of clinical variables and risk factors according to the occurrence of the primary outcome.

**Table 2:** – Univariate analysis of characteristics according to the primary outcome

Variables	Primary outcome		p
	Yes (n=57)	No (n=179)	
Age (years) - mean ± SD	71.3±13.5	59.2±15.9	<0.001
Age ≥ 65 years (%)	75.4	35.8	<0.001
Obesity (%)	28.1	17.9	0.072
Previous HF (%)	10.5	2.2	0.015
CKD (%)	12.3	2.8	0.010
SAH (%)	68.4	51.4	0.017
Diabetes (%)	49.1	27.9	0.003
Previous AMI (%)	7.0	5.6	0.450
PAD (%)	10.5	8.4	0.396
AF (%)	8.8	1.1	0.010
Previous stroke (%)	8.8	1.7	0.022
Tobacco use (%)	7.0	3.9	0.260
Dyslipidemia (%)	15.8	12.8	0.357
Mechanical ventilation (%)	87.0	12.4	<0.001
Vasopressor use (%)	70.4	10.6	<0.001
Renal replacement therapy (%)	35.2	2.9	<0.001
Myocardial injury (%)	80.7	13.4	<0.001
Troponin peak (ng/L) - median (IQR)	58 (13-276)	7 (4 – 10)	<0.001
D-dimer peak (ng/mL) - median (IQR)	7857 (4124-24121)	1327 (754-3087)	<0.001
SAPS3 - median (IQR)	52 (44 - 61)	39 (34 - 46)	<0.001
High cardiovascular risk (%)	70.2	40.2	<0.001

*Comparison of clinical and laboratory characteristics according to the primary outcome (in-hospital death). SD: standard deviation; HF: heart failure; CKD: chronic kidney disease; SAH: systemic arterial hypertension; AMI: acute myocardial infarction; PAD: Peripheral Artery Disease; AF: atrial fibrillation; IQR: interquartile range.*

In univariate analysis, several clinical characteristics were significantly associated with a higher prevalence of the primary outcome. Table 3 shows the univariate analysis related to the secondary outcome.

**Table 3:** – Univariate analysis of characteristics according to secondary outcome

Variables	Secondary outcome		p
	Yes (n=86)	No (n=138)	
Age (years) - mean ± DP	69.0±15.5	57.8±15.1	<0.001
Age ≥ 65 years (%)	67.0	31.7	<0.001
Obesity (%)	23.1	18.6	0.253
Previous HF (%)	7.7	2.1	0.041
CKD (%)	9.9	2.1	0.010
SAH (%)	69.2	46.9	0.001
Diabetes (%)	40.7	28.3	0.034
Previous AMI (%)	8.8	4.1	0.118
PAD (%)	12.1	6.9	0.130
AF (%)	5.5	1.4	0.080
Previous stroke (%)	6.6	1.4	0.039
Tobacco use (%)	5.5	4.1	0.427
Dyslipidemia (%)	14.3	13.1	0.471
Vasopressor use (%)	62.8	1.4	<0.001
Renal replacement therapy (%)	27.9	0.0	<0.001
D-dimer peak (ng/mL) - median (IQR)	6118 (3365-18433)	1030 (613-1880)	<0.001
SAPS3 - median (IQR)	37 (29-43)	50 (43-60)	<0.001
High cardiovascular risk (%)	62.6	37.9	<0.001

*Comparison of clinical and laboratory characteristics according to secondary outcome (composed of hospital death, myocardial injury, and need for mechanical ventilation). SD: standard deviation; HF: heart failure; CKD: chronic kidney disease; SAH: systemic arterial hypertension; AMI: acute myocardial infarction; PAD: Peripheral Artery Disease; AF: atrial fibrillation; IQR: interquartile range.*

Similar to the primary outcome, several characteristics were associated with the secondary outcome. In the analysis by risk group (HCR with myocardial injury, HCR without myocardial injury, non-HCR with myocardial injury, and non-HCR without myocardial injury), it was observed that the HCR with myocardial injury group had a higher mortality (57.9%), significantly higher than the groups without myocardial injury, but with no statistical difference compared to the non-HCR with myocardial injury group ( Figure 3 ).

**Figure 3:** – Occurrence of the primary outcome in the subgroups determined according to cardiovascular risk and troponin elevation. Comparison between groups performed with the chi-square test. HRC: high cardiovascular risk.

In the logistic regression adjusted for severity (SAPS3 score), the group of patients with HCR and myocardial injury had the highest risk of mortality, followed by the non-HCR group with myocardial injury ( Table 4 ).

**Table 4:** – Binomial logistic regression for the primary outcome.

Variable	OR	CI 95%	p
No HCR + no myocardial injury	Reference		
No HCR + myocardial injury	16.70	4.45-62.74	<0.001
HCR + no myocardial injury	2.06	0.56-7.56	0.2745
HCR + myocardial injury	40.38	11.78-138.39	<0.001
SAPS3	1.05	1.02-1.09	0.0023

*HRC: high cardiovascular risk.*

In the classification tree, for the primary outcome, myocardial injury was found to be the first classifying characteristic, followed by arterial hypertension. This classification model had an accuracy of 85.2% ( Figure 4 ).

**Figure 4:** – Classification tree for the primary outcome.

## Discussion

This study evaluated the impact of high cardiovascular risk in patients admitted to intensive care for COVID-19. This approach allowed for the inclusion of patients with a higher severity profile and a higher prevalence of cardiovascular risk factors. In this population, more than half of the patients had arterial hypertension and a third had diabetes. The high proportion of patients over 65 years of age (45.3%) is also noteworthy. The main finding of this study was the observation that patients with HCR had a significantly higher mortality only when associated with myocardial injury.

Most published studies only assessed the impact of cardiovascular risk factors on mortality from COVID-19, generating conflicting results. Di Castelnuovo et al.^8^ studied almost 4000 patients in an Italian multicenter study, using statistical analysis techniques based on machine learning^8^ . This study included older patients (54.8% over 65 years), but with a similar prevalence of arterial hypertension (48.8%) and a lower prevalence of diabetes (19%). The main predictors of in-hospital death were renal dysfunction, high CRP levels, and advanced age. No association was found with obesity, smoking, cardiovascular disease, and related comorbidities.

Collard et al.^9^ analyzed data from eight hospitals participating in the CovidPredict cohort in Germany.^9^ For the analysis of cardiovascular risk factors, they evaluated the use of antihypertensive, antidiabetic, and lipid-lowering drugs. The study included 1,604 patients with a mean age of 66 years; 46% were hypertensive and 25.7% diabetic. It was observed that patients with more than one cardiovascular risk factor had a 52% higher 3-week mortality, regardless of gender and age. Furthermore, the use of two or more antihypertensive or antidiabetic, or lipid-lowering drugs was associated with a worse prognosis in patients with COVID-19. Our study found a similar result, demonstrating a progressive increase in hospital mortality as the number of risk factors increases.

Silverio et al.^10^ performed a meta-analysis that included 18,300 patients.^10^ In the univariate analysis, an association between in-hospital death and age, diabetes, and hypertension was observed. However, in multivariate regression, only diabetes and older age were associated with hospital death.

Only one study used a data analysis strategy similar to the present study. Guo et al.^11^ analyzed 187 patients in Wuhan (origin of the pandemic), aiming to assess the association of underlying cardiovascular disease and myocardial injury with fatal outcomes in patients with COVID-19.^11^ This population was younger (mean age=58.5 years), with a lower prevalence of hypertension (32.6%) and diabetes (15.0%). In the statistical analysis of this study, data related to cardiovascular risk were not evaluated, but regarding established cardiovascular disease, which was defined by the presence of hypertension, coronary artery disease and, cardiomyopathy. Patients with established cardiovascular disease and myocardial injury had a mortality of 69.44%, while patients without cardiovascular disease but with myocardial injury had a mortality of 37.5%. These results are similar to those described in this article, although different classification criteria and statistical techniques were used.

Thus, we observed that no study published so far aimed to study patients characterized as having high cardiovascular risk, which represented 47.4% of this sample. Troponin, a marker of myocardial injury, demonstrated its prognostic importance in previous studies^4 , 11^ and, in this study, in the classification tree, it was the first prognostic marker of in-hospital mortality. In the subgroup of patients who did not present myocardial injury, arterial hypertension was the comorbidity significantly associated with in-hospital death.

In logistic regression, the SAPS3 score, a severity score in intensive care performed on admission,^12^ was used to adjust for potential confounders in the analysis of subgroups according to cardiovascular risk and myocardial injury. After adjustment, it was observed that patients with high cardiovascular risk and myocardial injury had a 40-fold higher risk of in-hospital death when compared to patients without high risk and normal troponin, regardless of the severity presented upon admission. In patients with myocardial injury, but without high cardiovascular risk, a high risk of mortality was observed (OR 16.70; 95% CI 4.45-62.74), but with a lower magnitude when compared to patients with a high cardiovascular risk and myocardial injury. By contrast, in patients without myocardial injury, the high cardiovascular risk did not significantly impact in-hospital death.

This study has some limitations that are inherent to a retrospective study. All data were evaluated through the verification of electronic medical records, and it was not possible to confirm data or additional questions to the patient or family members. In addition, not all patients underwent echocardiography or BNP measurements, which are important information in a study that assesses the cardiovascular impact of COVID-19. Furthermore, the small number of patients in the study limits the statistical analysis and conclusions drawn from these results.

Despite the limitations, this is the first study that specifically analyzes the population at high cardiovascular risk in patients admitted to the intensive care unit for COVID-19.

## Conclusions

In patients hospitalized in intensive care for COVID19, the presence of high cardiovascular risk impacts in-hospital mortality only in patients with elevated troponin levels.
